# Sex difference for the risk of amputation in diabetic patients: A systematic review and meta-analysis

**DOI:** 10.1371/journal.pone.0243797

**Published:** 2021-03-11

**Authors:** Lei Fan, Xue-Jian Wu

**Affiliations:** 1 Department of Orthopedic Surgery, The First Affiliated Hospital of Zhengzhou University, Zhengzhou, China; 2 Department of Orthopedic Surgery, People’s Hospital of Zhengzhou University, Zhengzhou, China; Universita degli Studi di Roma Tor Vergata, ITALY

## Abstract

The risk of amputation is a sequelae of diabetic foot ulceration, which are significantly increased in diabetic patients and caused huge morbidly and mortality. However, whether the risk amputation in diabetic patients are differing in male and female remains inconclusive. We therefore conducted a systematic review and meta-analysis to assess the sex difference for the risk of amputation in diabetic patients. We systematically searched PubMed, EmBase, and the Cochrane library to identify eligible study from their inception up to November 2020. The diagnostic value of male patients on subsequent amputation risk were assessed by using sensitivity, specificity, positive and negative likelihood ratio (PLR and NLR), diagnostic odds ratio (DOR), and area under the receiver operating characteristic curve (AUC). Twenty-two studies recruited a total of 33,686,171 diabetic patients were selected for quantitative analysis. The risk of amputation in male diabetic patients was greater than female diabetic patients (DOR: 1.38; 95%CI: 1.13–1.70; *P*<0.001). The sensitivity and specificity for male diabetic patients on the risk of amputation were 0.72 (95%CI: 0.72–0.73), and 0.51 (95%CI: 0.51–0.51), respectively. Moreover, the PLR and NLR of male diabetic patients for predicting amputation were 1.13 (95%CI: 1.05–1.22), and 0.82 (0.72–0.94), respectively. Furthermore, the AUC for male diabetic patients on amputation risk was 0.56 (95%CI: 0.48–0.63). This study found male diabetic patients was associated with an increased risk of amputation than female diabetic patients, and the predictive value of sex difference on amputation risk in diabetic patients was mild.

## Introduction

Diabetic foot is a common complication in diabetic patients, which consisted the lesions in deep tissues in the lower limb and caused neurological disorders and peripheral vascular disease [1]. The prevalence of diabetic foot ulcers ranged from 19–34 percent in diabetic patients, and the annual incidence rates for diabetic foot ulcers in general diabetic patients nearly 6.3 percent [2, 3]. Study have already illustrated the complications could induce serious public health problem, and caused most common cause for hospital ingress, amputation, and mortality in diabetic patients [4]. Moreover, there was nearly USD 727 billion could spent for diabetic patients aged 20–79 years based on data from the International Diabetes Federation [5]. Nearly two-thirds of diabetic foot ulcers could heal, and up to 28% of patients should treated with lower extremity amputation [6–8].

Major amputation often causes substantial functional disability, and associated with significant morbidity and mortality across world. Moreover, patients after major amputation always needs various type of prosthesis to walk by itself [9]. Several systematic reviews and meta-analyses have already conducted to identify potentially risk factors for amputation in patients resented diabetic foot ulcers [10–12]. However, whether the risk of major amputation in diabetic patients are differing in male and female remains inconclusive, which needed further clarifying to determine the diabetic population at high risk for further amputation. Therefore, the current systematic review and meta-analysis was conducted to assess the sex difference for the risk of amputation in diabetic patients.

## Materials and methods

### Data sources, search strategy, and selection criteria

This study was conducting and reporting following the Preferred Reporting Items for Systematic Reviews and Meta-Analysis Statement [13]. Study reported the amputation occurred according to male and female diabetic patients was eligible in this study, and published language or status were not restricted. The electronic searches were performed in the databases of PubMed, EmBase, and the Cochrane library throughout November 2020, and the following terms were used as text word or Medical Subject Heading: "diabetic foot" and "amputation" (S1 File). Moreover, the reference lists of relevant review and original article were also reviewed by manually to identify any new study met the inclusion criteria.

Two reviewers independently performed literature search and study selection following a standardized process, and inconsistencies were settled by discussion after reviewing the original article. The details of inclusion criteria are listed as follows: (1) Patients: all of patients were diagnosed with diabetes, irrespective diabetes type; (2) Exposure: male and female; (3) Outcome: the incidence of major amputation; and (4) Study design: we did not restricted the study design, including prospective and retrospective studies.

### Data collection and quality assessment

Two reviewers independently abstracted the following items: first authors’ name, publication year, country, study design, number of amputations, sample size, number of male and female, age, diabetes type, diabetes duration, HbA1c, setting, follow-up duration, and amputation cases in male and female groups. Then the methodological quality of individual study was independently assessed by 2 reviewers using the Newcastle-Ottawa Scale (NOS), which on the basis of selection (4 items), comparability (1 item), and outcome (3 items) [14]. Any disagreement between 2 reviewers for data collection and quality assessment was settled by an additional author referring to the full-text of included studies.

### Statistical analysis

The diagnostic odds ratio (DOR) and 95% confidence intervals (CIs) was firstly applied to assess the sex difference for the risk of amputation in diabetic patients, and the pooled analysis was calculated using the random-effects model [15, 16]. After this, the pooled predictive values (sensitivity, specificity, positive likelihood ratio [PLR], negative likelihood ratio [NLR], and the area under the receiver operating characteristic curve [AUC]) were assessed on the basis of the prevalence of amputation in male and female diabetic patients [17]. After this, the heterogeneity across studies were assessed by using *I*^*2*^ and Q statistic, and the significant heterogeneity was defined as *I*^*2*^ > 50.0% or *P* < 0.10 [18, 19]. Subgroup analyses for diagnostic parameters were also conducted based on study design, age, diabetes type, and HbA1c level. The funnel plot and Deeks’ asymmetry test was applied to assess any potentially publication bias [20]. The *P* value for all pooled results are 2-sided, and the inspection level was 0.05. All of analyses in our study was conducted by using software STATA (version 10.0; Stata Corporation, TX, USA).

## Results and discussion

### Literature search

**Fig 1:**
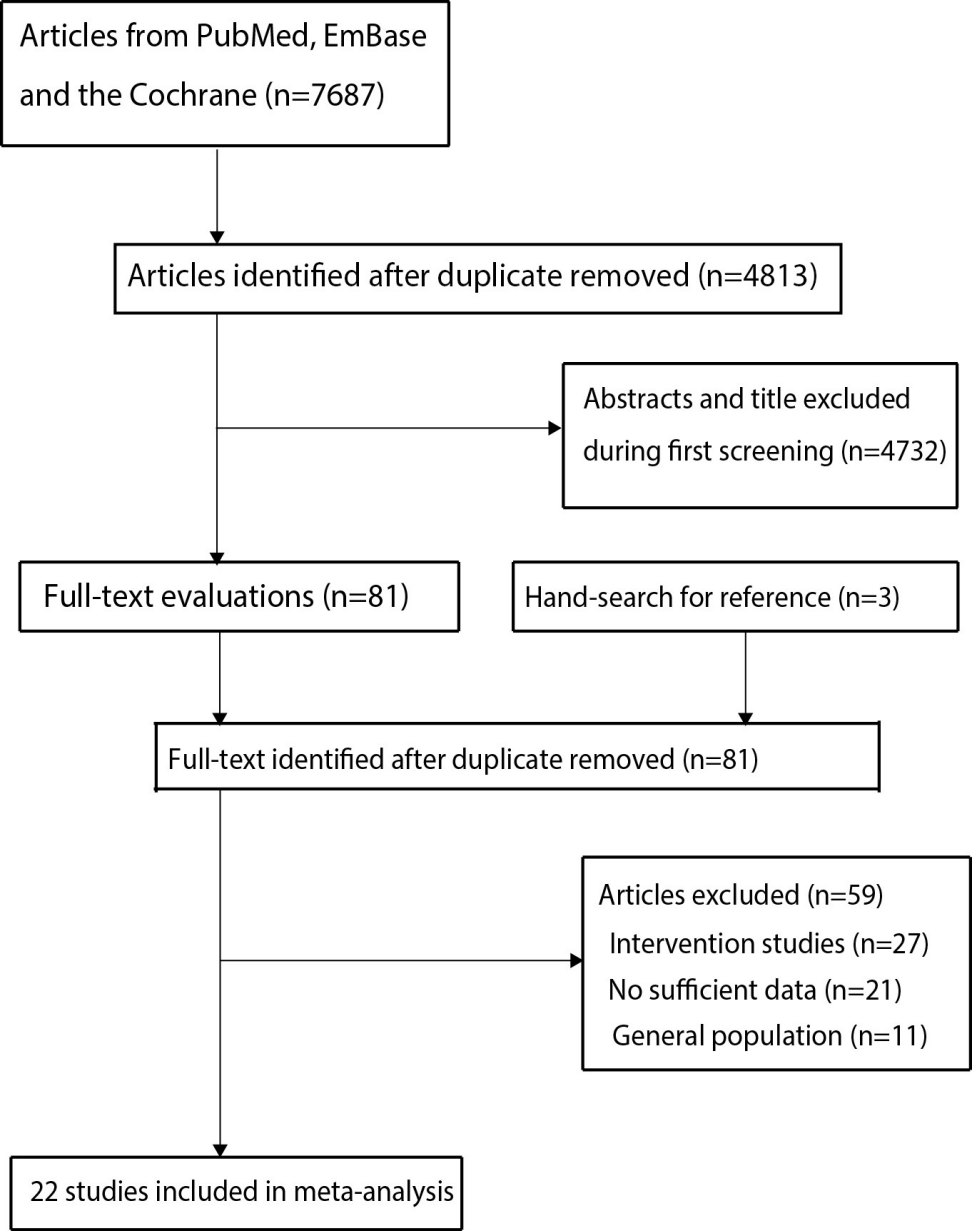
The PRISMA flowchart for the study selection process.

A total of 7,687 records were identified by initial electronic searches in PubMed, EmBase, and the Cochrane library. After this, the 4,813 articles were retained after duplicate titles were removed. Then 4,732 studies were excluded because of these studies reported irrelevant titles. The remaining 81 studies were retrieved for full-text evaluations, and 59 studies were excluded because of: Intervention studies (n = 27), no sufficient data (n = 21), and studies included general population (n = 11). Therefore, the remaining 22 studies were selected for final meta-analysis [21–42], and the details regarding study selection are presented in Fig 1.

### Study characteristics

The characteristics of included studies and patients are presented in Table 1. Of included studies, 8 studies were designed as prospective cohort, while the remaining 14 studies were designed as retrospective design. The number of amputation events for included studies ranged from 10 to 14,627, and the sample size ranged from 37 to 27,562,858. Three studies were conducted in Eastern Asia, 9 studies were conducted in Central Asia, 4 studies were conducted in America, 4 studies were conducted in Europe or Australia, and the remaining 2 studies were conducted in Africa. The quality assessment for individual study was applied the NOS, 7 studies with 9 stars, 7 studies with 8 stars, and the remaining 8 studies with 7 stars (S1 Table).

**Table 1:** The baseline characteristics of included studies and patients.

Study	Country	Study design	No. of amputations	Sample size	Male/female	Age (years)	DM type	DM duration	HbA1c (%)	Setting	Follow-up
**Armstrong 1997 [21]**	USA	Retrospective	31	77	51/26	52.5	NA	NA	NA	University Hospital	NA
**Lin 2010 [22]**	China	Retrospective	24	90	47/43	69.7	T2DM	15.2 years	9.32	NA	NA
**Akinci 2011 [23]**	Turkey	Prospective	70	165	109/56	60.2	T2DM (95.8%)	15.0 years	9.50	NA	6.0 months
**Aziz 2011 [24]**	Singapore	Prospective	55	100	51/49	59.8	T2DM	> 5.0 years	NA	University Hospital	2.0 years
**Tunccan 2012 [25]**	Turkey	Retrospective	12	71	46/25	60.6	NA	NA	NA	Infectious Diseases Clinic	NA
**Ulcay 2014 [26]**	Turkey	Retrospective	22	37	27/10	65.0	NA	17.2 years	8.40	NA	1.0 year
**Saltoglu 2015 [27]**	Turkey	Retrospective	126	455	310/145	61.3	T2DM (99.3%)	15.4 years	NA	Multicentre	3.0–6.0 months
**Pickwell 2015 [28]**	Netherlands	Prospective	159	575	359/216	65.6	NA	NA	NA	Diabetic Foot Center	1.0 year
**Tabur 2015 [29]**	Turkey	Retrospective	10	55	27/28	60.0	T2DM	11.1 years	10.40	Endocrinology Department	NA
**Quilici 2016 [30]**	Brazil	Prospective	61	100	68/32	62.0	T2DM (99.0%)	NA	NA	Vascular Surgery Clinic	NA
**Uysal 2017 [31]**	Turkey	Prospective	126	379	256/123	62.4	T2DM (95.8%)	15.0 years	8.30	Diabetic Foot Council	NA
**Cervantes-García 2017 [32]**	Mexico	Prospective	45	100	60/40	51.2	T2DM	10.0 years	NA	Emergency Department	NA
**Ferreira 2018 [33]**	Portugal	Retrospective	48	479	294/185	68.0	T2DM (90.8%)	15.0 years	7.80	Diabetic Foot Clinic	1.0 year
**Musa 2018 [34]**	Saudi Arabia	Prospective	33	82	55/27	60.0	NA	8.5 years	4.80	King Abdul Aziz Armed Forces Hospital	NA
**Khalfallah 2018 [35]**	Tunisia	Retrospective	95	430	319/111	60.5	NA	NA	NA	Charles Nicolle hospital	NA
**Peled 2019 [36]**	Israel	Retrospective	229	418	311/107	64.8	T2DM (92.6%)	NA	NA	Academic tertiary hospital	NA
**Guo 2019 [37]**	China	Retrospective	59	470	294/176	63.3	NA	9.2 years	8.26	Third Xiangya Hospital	NA
**Jeyaraman 2019 [38]**	Australia	Retrospective	263	513	322/191	56.1	T2DM (93.6%)	7.0 years	NA	Multidisciplinary Foot Clinic	5.8 years
**Ugwu 2019 [39]**	Nigeria	Prospective	119	336	185/151	55.9	T2DM (96.1%)	8.5 years	9.60	Multicentre	1.0 year
**Sayiner 2019 [40]**	Turkey	Retrospective	143	400	256/144	> 18.0	T2DM	NA	NA	Endocrinology and Metabolism of the Faculty of Medicine of Gaziantep University	NA
**Aziz 2020 [41]**	Austria	Retrospective	2,165	27,562,858	13,358044/14,204,814	73.0	T2DM (83.3%)	NA	NA	Austrian Health Insurance database	NA
**Gandhi 2020 [42]**	USA	Retrospective	14,627	6,117,981	3,180,967/2,937,014	56.5	T2DM	NA	NA	Truven Health MarketScan database	NA

### DOR

**Fig 2:**
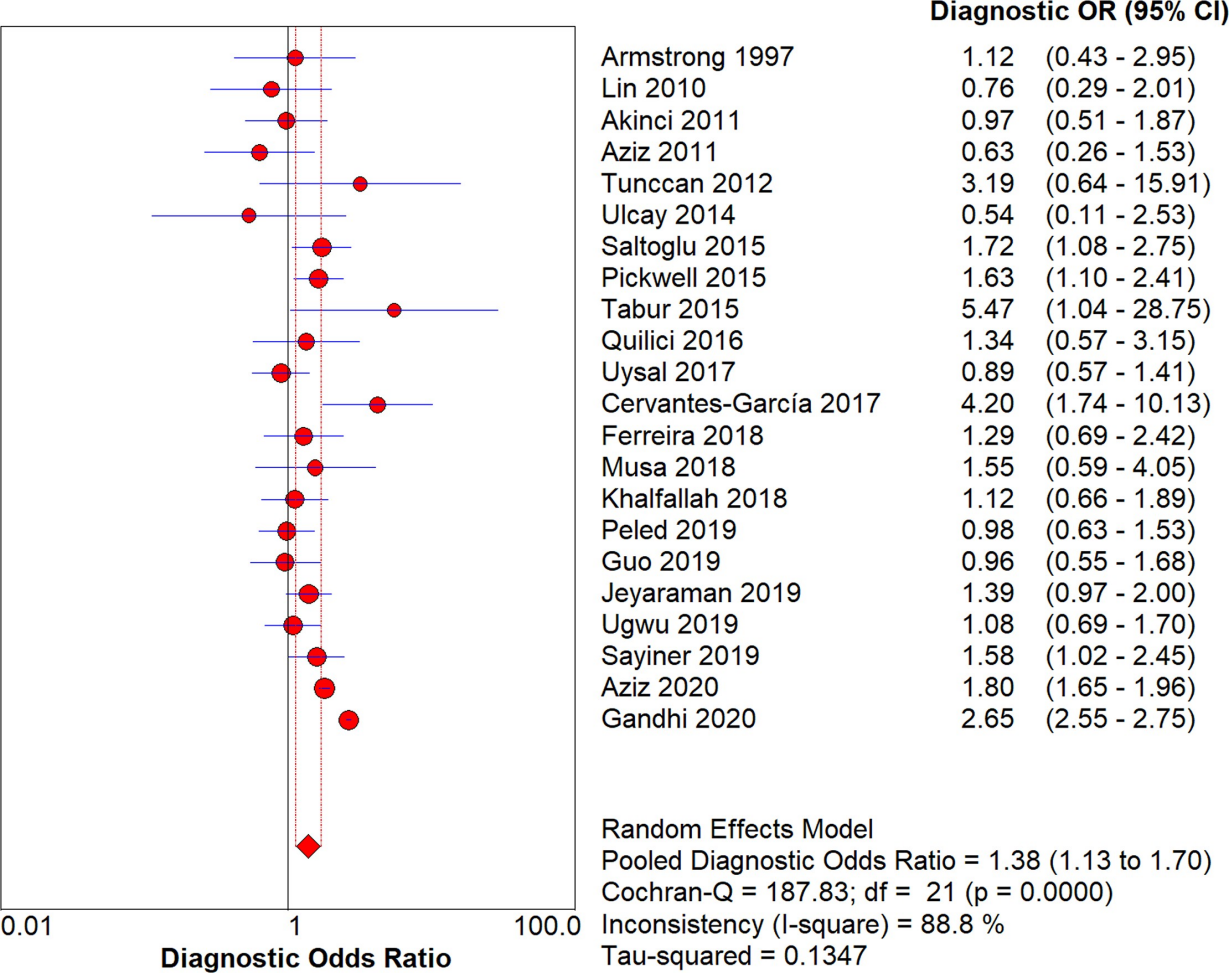
The summary DOR of male on subsequent amputation in diabetic patients.

After pooling all included studies, we noted male diabetic patients was associated with an increased risk of amputation as compared with female diabetic patients (DOR: 1.38; 95%CI: 1.13–1.70; *P*<0.001; Fig 2), and significant heterogeneity was detected across included studies (*I*^*2*^ = 88.8%; *P*<0.001). Subgroup analysis found the significant sex difference was detected when study designed as retrospective cohort, irrespective age of patients, irrespective diabetes type, and the level of HbA1c was not reported (Table 2).

**Table 2:** Subgroup analyses.

Parameters	Factors	Subgroup	Effect estimate and 95%CI	*I*^*2*^ (%)	*P* value for Q statistic
**Sensitivity**	Study design	Prospective	0.66 (0.62–0.70)	58.1	0.019
		Retrospective	0.73 (0.72–0.73)	90.0	< 0.001
	Age (years)	≥ 60.0	0.65 (0.64–0.67)	59.2	0.002
		< 60.0	0.74 (0.73–0.74)	84.2	< 0.001
	DM type	T2DM	0.72 (0.72–0.73)	90.9	< 0.001
		Not reported	0.71 (0.66–0.75)	0.0	0.537
	HbA1c (%)	≥ 9.00	0.60 (0.53–0.67)	21.2	0.283
		< 9.00	0.66 (0.60–0.71)	0.0	0.847
		Not reported	0.73 (0.72–0.73)	91.0	< 0.001
**Specificity**	Study design	Prospective	0.40 (0.37–0.43)	61.8	0.011
		Retrospective	0.51 (0.51–0.51)	99.9	< 0.001
	Age (years)	≥ 60.0	0.52 (0.52–0.52)	95.8	< 0.001
		< 60.0	0.48 (0.48–0.48)	64.5	0.010
	DM type	T2DM	0.51 (0.51–0.51)	99.9	< 0.001
		Not reported	0.36 (0.33–0.38)	71.3	0.002
	HbA1c (%)	≥ 9.00	0.43 (0.39–0.48)	63.9	0.040
		< 9.00	0.37 (0.34–0.40)	36.5	0.178
		Not reported	0.51 (0.51–0.51)	100.0	< 0.001
**PLR**	Study design	Prospective	1.09 (0.97–1.23)	51.0	0.046
		Retrospective	1.15 (1.06–1.25)	91.2	< 0.001
	Age (years)	≥ 60.0	1.10 (1.01–1.20)	77.1	< 0.001
		< 60.0	1.19 (1.02–1.39)	84.6	< 0.001
	DM type	T2DM	1.15 (1.06–1.25)	91.2	< 0.001
		Not reported	1.09 (1.00–1.19)	13.8	0.324
	HbA1c (%)	≥ 9.00	1.10 (0.87–1.38)	58.2	0.066
		< 9.00	1.00 (0.91–1.11)	0.0	0.649
		Not reported	1.18 (1.09–1.28)	91.2	< 0.001
**NLR**	Study design	Prospective	0.88 (0.72–1.07)	50.7	0.048
		Retrospective	0.79 (0.67–0.93)	91.0	< 0.001
	Age (years)	≥ 60.0	0.85 (0.75–0.96)	41.9	0.044
		< 60.0	0.76 (0.57–1.01)	89.2	< 0.001
	DM type	T2DM	0.81 (0.69–0.95)	92.1	< 0.001
		Not reported	0.85 (0.72–1.00)	0.0	0.540
	HbA1c (%)	≥ 9.00	1.10 (0.87–1.38)	58.2	0.066
		< 9.00	0.99 (0.82–1.20)	0.0	0.699
		Not reported	0.75 (0.64–0.88)	91.4	< 0.001
**DOR**	Study design	Prospective	1.26 (0.91–1.73)	52.1	0.041
		Retrospective	1.47 (1.16–1.86)	90.2	< 0.001
	Age (years)	≥ 60.0	1.29 (1.04–1.60)	56.8	0.004
		< 60.0	1.57 (1.02–2.41)	86.9	< 0.001
	DM type	T2DM	1.43 (1.14–1.81)	91.2	< 0.001
		Not reported	1.30 (1.01–1.66)	0.0	0.491
	HbA1c (%)	≥ 9.00	0.97 (0.79–1.20)	3.9	0.374
		< 9.00	1.01 (0.76–1.35)	0.0	0.681
		Not reported	1.59 (1.27–2.00)	90.4	< 0.001
**AUC**	Study design	Prospective	0.49 (0.36–0.61)	-	-
		Retrospective	0.61 (0.52–0.69)	-	-
	Age (years)	≥ 60.0	0.62 (0.60–0.64)	-	-
		< 60.0	0.46 (0.37–0.54)	-	-
	DM type	T2DM	0.55 (0.46–0.64)	-	-
		Not reported	0.55 (0.41–0.70)	-	-
	HbA1c (%)	≥ 9.00	0.50 (0.39–0.62)	-	-
		< 9.00	0.56 (0.33–0.78)	-	-
		Not reported	0.59 (0.51–0.68)	-	-

### Diagnostic parameters

**Fig 3:**
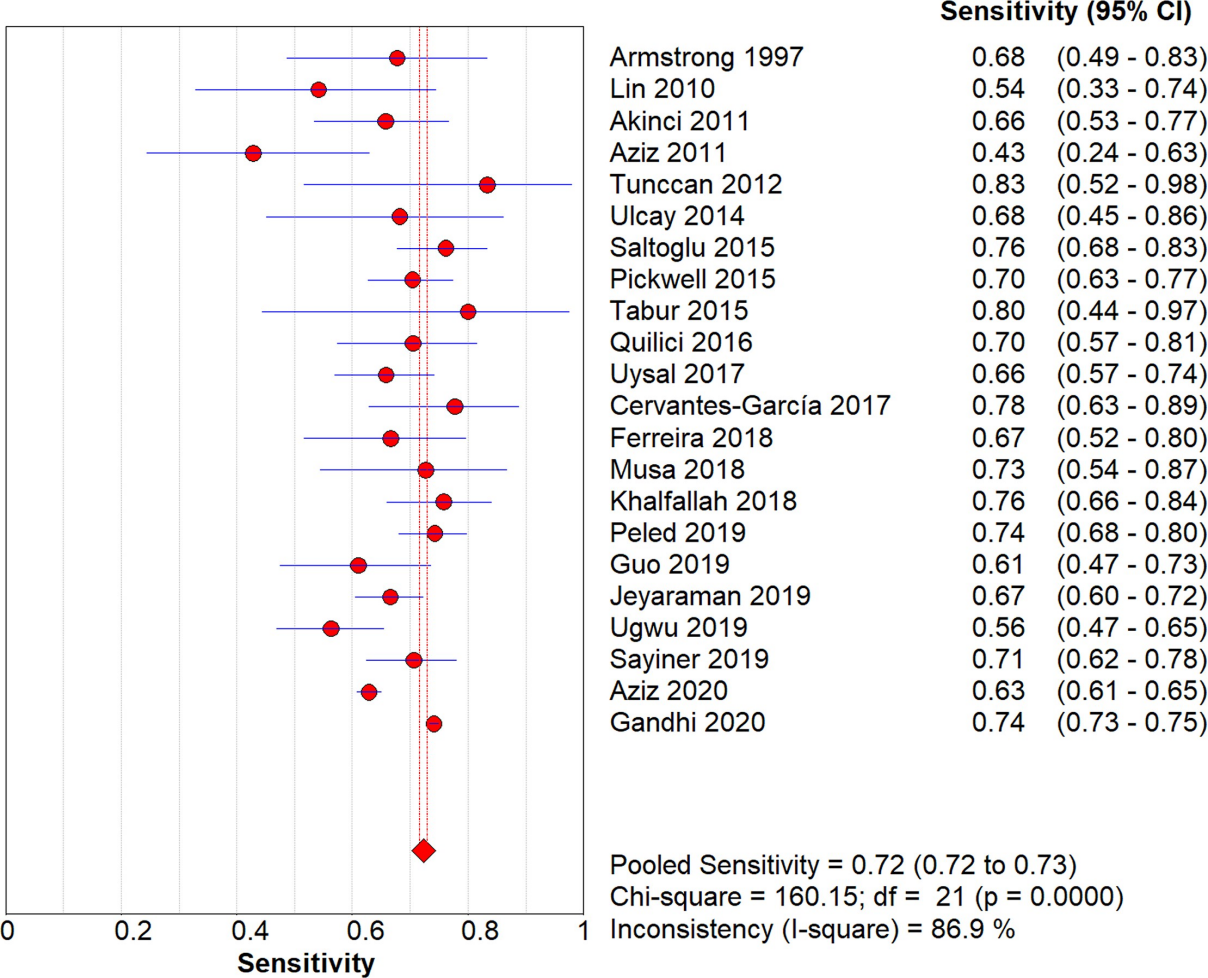
The summary sensitivity for male on subsequent amputation in diabetic patients.

**Fig 4:**
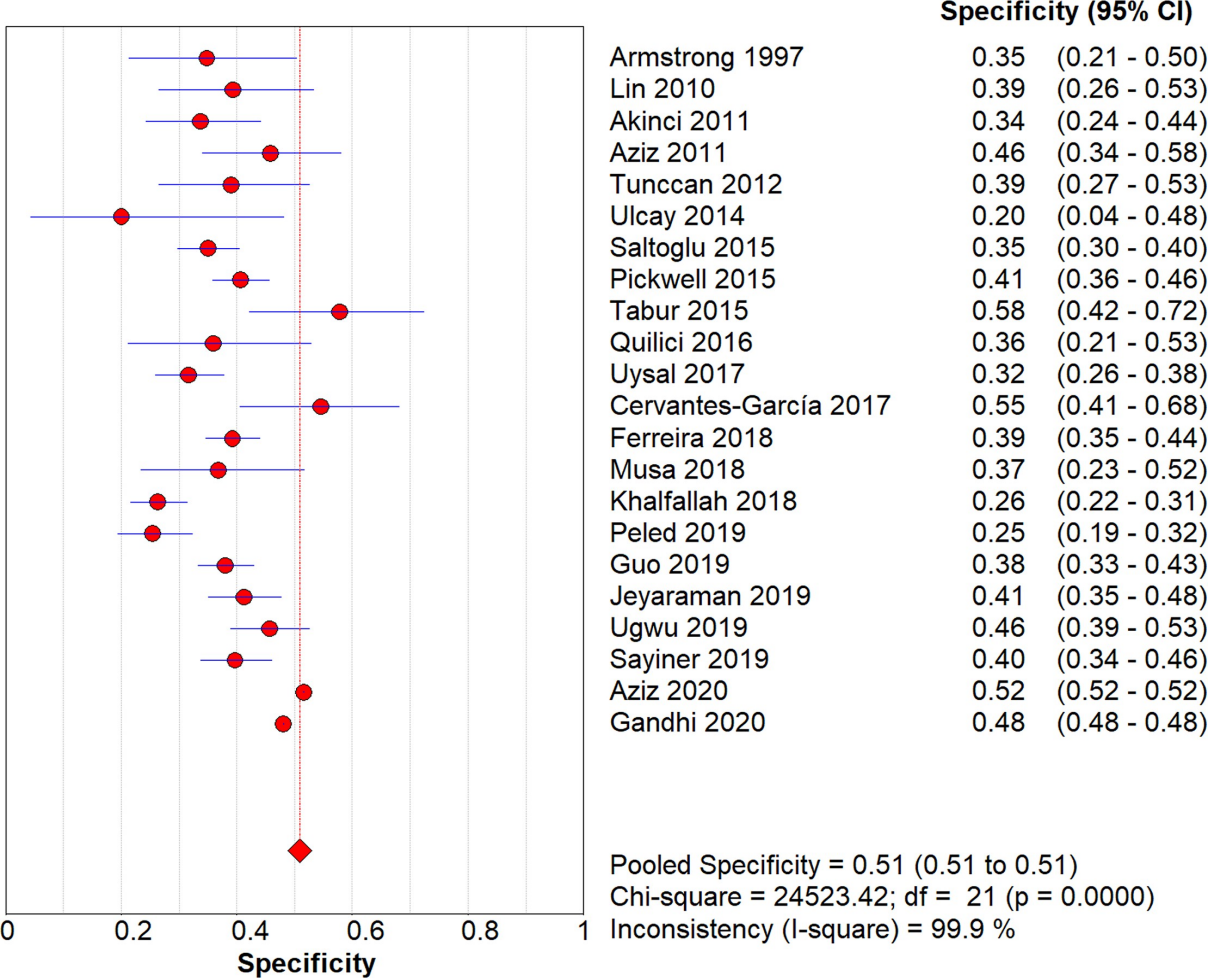
The summary specificity for male on subsequent amputation in diabetic patients.

**Fig 5:**
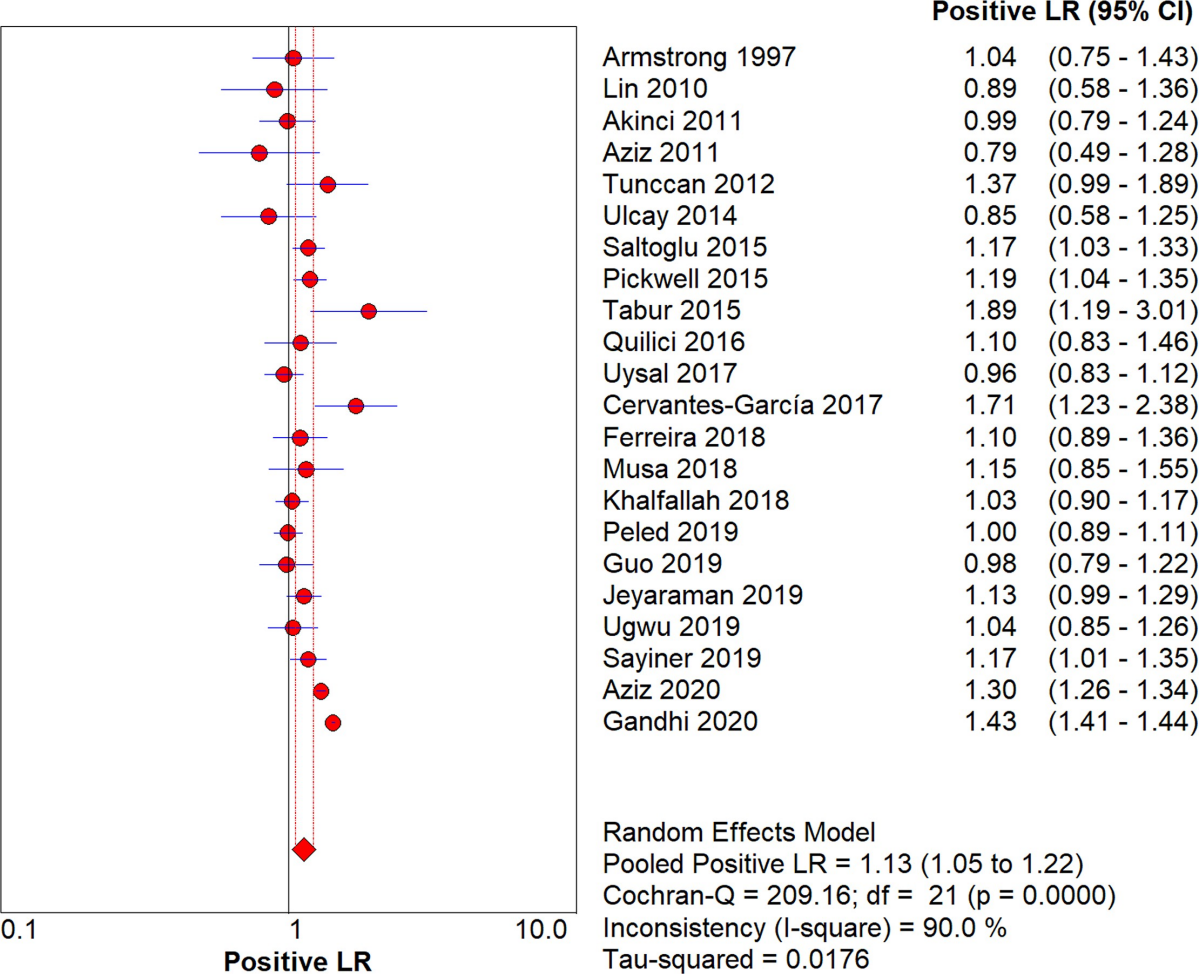
The summary PLR for male on subsequent amputation in diabetic patients.

**Fig 6:**
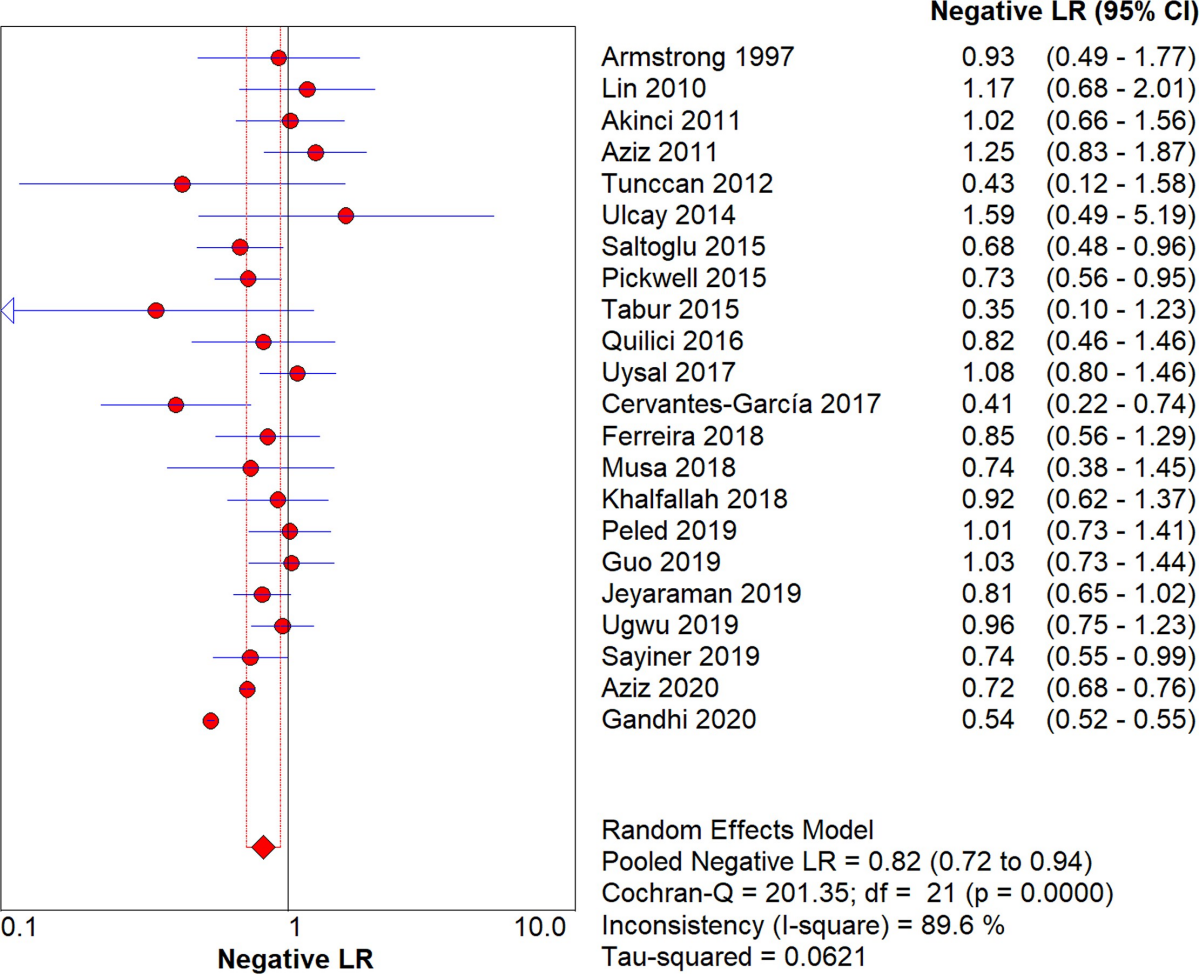
The summary NLR for male on subsequent amputation in diabetic patients.

**Fig 7:**
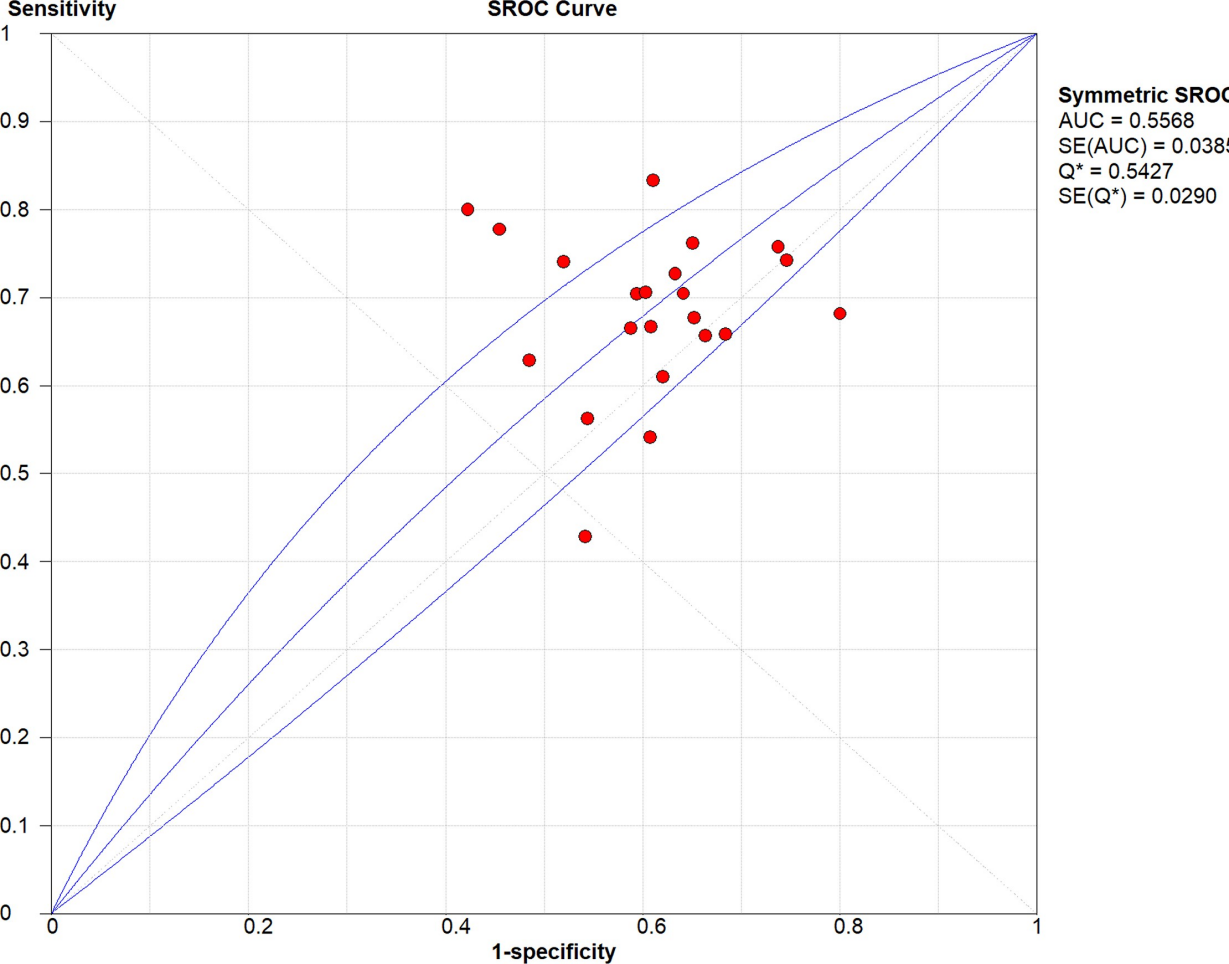
The summary SROC for male on subsequent amputation in diabetic patients.

**Fig 8:**
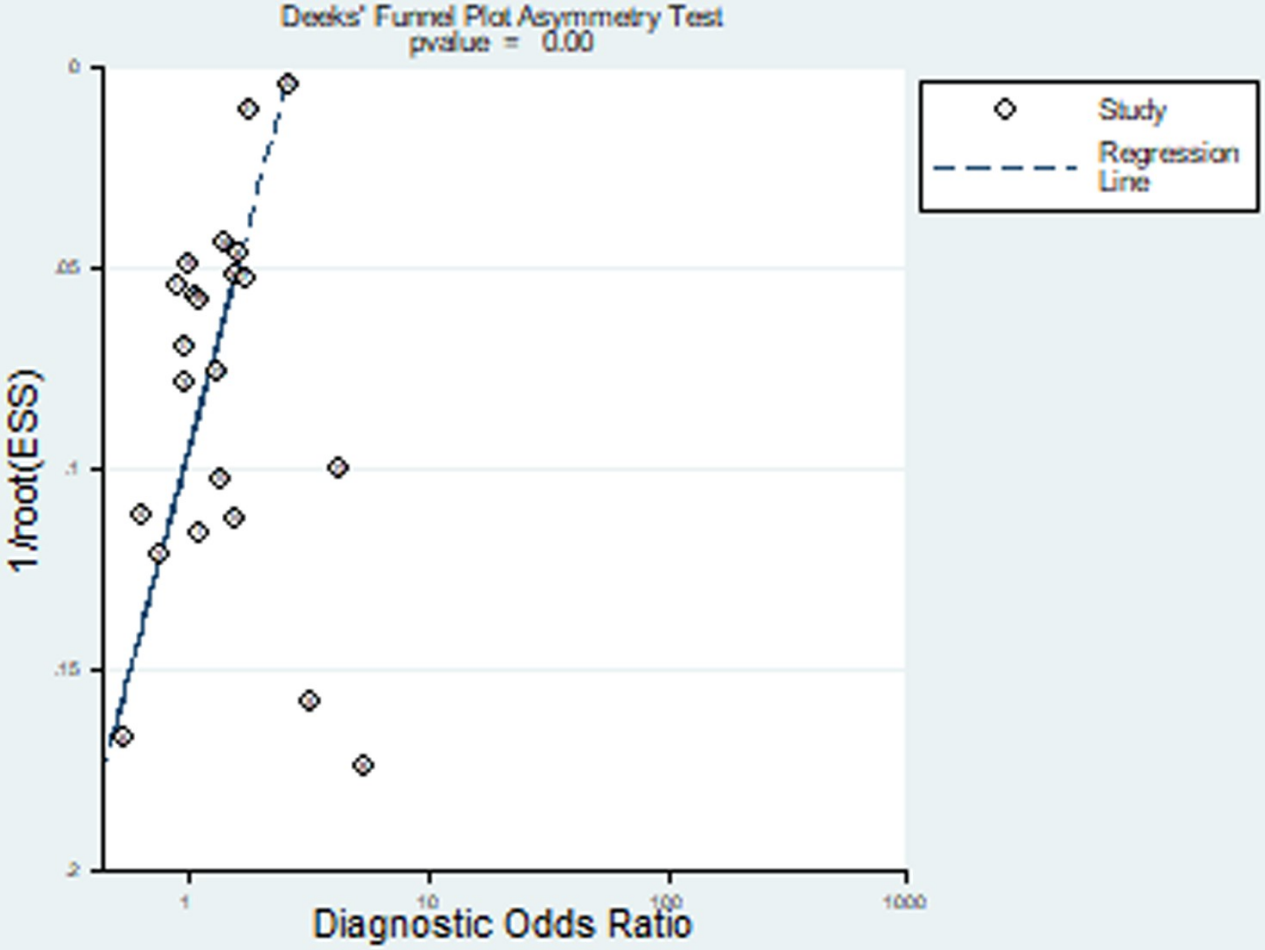
Publication bias.

After pooling all studies, we noted the pooled sensitivity and specificity for male patients on amputation risk were 0.72 (95%CI: 0.72–0.73; Fig 3), and 0.51 (95%CI: 0.51–0.51; Fig 4), respectively. The sensitivity was associated with statistically significant in all subgroups, while the specificity was associated with statistically significant if study designed as retrospective cohort, age of patients > 60.0 years, type 2 diabetes, and the level of HbA1c was not reported (Table 2). Moreover, we noted the pooled PLR and NLR for male patients on amputation risk were 1.13 (95%CI: 1.05–1.22; Fig 5), and 0.82 (0.72–0.94; Fig 6), respectively. Subgroup analyses found the pooled PLR were associated with statistically significant when pooled study designed as retrospective cohort, irrespective age of patients, or diabetes type, and the level of HbA1c was not reported (Table 2). Similarly, the pooled NLR with statistically significant when pooled study designed as retrospective cohort, age of patients > 60.0 years, type 2 diabetes, or the level of HbA1c was not reported (Table 2). Finally, the AUC for male patients on subsequent amputation was 0.56 (95%CI: 0.48–0.63; Fig 7), which was not associated with statistically significant. The results of subgroups indicated significant predictive value when pooled study designed as retrospective cohort, age of patients > 60.0 years, or the level of HbA1c was not reported (Table 2; Fig 8).

### Publication bias

The publication bias could not rule out by review funnel plot, and significant publication bias was seen by Deeks’ test (*P*<0.01).

### Significance and impacts

Numerous studies have already conducted to identify any potential risk factors for amputation risk in diabetic patients [10–12]. However, whether the sex difference was existed for amputation risk in diabetic patients remains controversial. The current quantitative analysis involved 33,686,171 diabetic patients from 22 studies and found male diabetic patients was associated with an increased risk of amputation than female diabetic patients, while the predictive value of sex difference was mild. Moreover, subgroup analysis found the significant sex difference mainly detected in the groups of study designed as retrospective cohort, irrespective age of patients, or diabetes type, and the level of HbA1c was not reported.

Several systematic review and meta-analyses have already conducted to identify potentially risk factors for amputation in patients with diabetic foot ulcer. Shin et al contained 10 studies and found hypertension, ischemic heart disease, cerebrovascular disease, and peripheral vascular disease were associated with an increased risk of major amputation [10]. A meta-analysis conducted by Wang et al found ulcer reaching bone, gangrene, hindfoot position, decreased ankle-brachial index, infection, and peripheral arterial disease could induce excess risk of major amputation in diabetic foot patients [11]. Sen et al conducted a meta-analysis of 25 studies and given a comprehensive risk profiles for lower extremity amputation for patients with diabetic foot infections [12]. However, the stratified analyses according to study and patients’ characteristics were not illustrated. Moreover, the sex difference for the risk of amputation in diabetic patients remains inconclusive. We therefore conducted a systematic review and meta-analysis to assess potential sex difference for the risk of amputation in diabetic patients.

The summary result of this study found male versus female diabetic patients was associated with an increased risk of amputation. However, mostly included studies did not found significant difference between male and female for the risk of amputation, while several studies reported similar results. A study conducted by Saltoglu et al found 76% of patients with amputation were male, while only 65% of patients without amputation were male [27]. Pickwell et al found male patients was associated with an increased risk of amputation excluding lesser toes as compared with female [28]. Tabur et al found the prevalence of male patients in lower extremity amputation group was 80%, while this prevalence in non-lower extremity amputation group was 42.2% [29]. Cervantes-García conducted a prospective study of 100 patients with infected diabetic foot ulcers and found 35 of 45 patients in amputation group was male, while just 25 of 55 patients in non-amputation group was male [32]. Sayiner et al conducted a retrospective study and found male patients was associated with an increased risk of amputation as compared with female patients [40]. Austrian Health Insurance database found male sex was associated with an increased risk of lower extremity amputation using adjusted negative binomial regression [41]. The Truven Health MarketScan database suggested male and older diabetic patients with high risk of lower limb amputations [42]. The potential reason for this could be the predisposing factor for the risk of amputation was not fully illustrated [43]. Moreover, the behavior in male and female are differences, which could explain the sex difference for the risk of amputation. Furthermore, male patients always under more physical and social pressure than female, which could be as a reason to force male feel healthy and strong than female. In addition, the hormonal protective role of estrogen could lead to differences in immune system function between male and female [44, 45]. Finally, the biological factors of diabetic foot ulcer, peripheral vascular disease, coronary artery disease, and peripheral neuropathy might accounts for the significant sex difference for the amputation rates [46, 47].

The predictive vale of male on subsequent amputation in diabetic patients were mild, and stratified analyses indicated the high predictive value were observed in the groups of studies designed as retrospective cohort, irrespective age of patients, or diabetes type, and the level of HbA1c was not reported. Several potential reasons could explained the above results: (1) the results from retrospective studies might induce overestimate effect estimates owing the uncontrolled selection and recall biases; (2) elderly patients always presented more serious disease, and event rates of amputation were higher than younger patients, caused the result with statistically significant was easily obtained. However, the pooled results for younger patients was associated with statistically significant was attributed to the result from Truven Health MarketScan database; (3) although mostly studies did not reported diabetes type, while the type 2 diabetes were accounts for predominent population; and (4) the HbA1c level could reflect the disease control, and affect the further risk of amputation.

Several shortcomings of this study should be mentioned. Firstly, mostly included studies designed as retrospective cohort, and the selection and recall biases were inevitable. Secondly, the characteristics of patients were not adjusted, which could affect the further amputation risk in diabetic patients. Thirdly, stratified analyses analyses according to patients’ characteristics were restricted owing to the analysis based on pooled data. Fourthly, the analysis based on published articles, while unpublished data were not available, and the publication bias was inevitable.

## Conclusions

In conclusion, this study found male diabetic patients was associated with an increased risk of amputation than female diabetic patients, while the predictive value for male on amputation risk in diabetic patients were mild. Moreover, the findings of this study needed further verified in further large-scale prospective cohort studies.

## Supporting information

S1 File Search strategy. (DOCX)

S1 Table The Newcastle-Ottawa scale of individual study. (DOCX)
