# Supplementary material for: Sex difference for the risk of amputation in diabetic patients: A systematic review and meta-analysis
Source: PLoS One. 2021 Mar 11;16(3):e0243797. doi: 10.1371/journal.pone.0243797 (PMC7951841; doi:10.1371/journal.pone.0243797)
Supplement: S1 File — (DOCX) [file pone.0243797.s001.docx]

**Search strategy:**

("Diabetic Foot/complications"[Mesh] OR "Diabetic Foot/epidemiology"[Mesh] OR "Diabetic Foot/ethnology"[Mesh] OR "Diabetic Foot/etiology"[Mesh]) AND ("Amputation/epidemiology"[Mesh] OR "Amputation/etiology"[Mesh] OR "Amputation/prevention and control"[Mesh])
