# Supplementary material for: Sex difference for the risk of amputation in diabetic patients: A systematic review and meta-analysis
Source: PLoS One. 2021 Mar 11;16(3):e0243797. doi: 10.1371/journal.pone.0243797 (PMC7951841; doi:10.1371/journal.pone.0243797)
Supplement: S1 Table — (DOCX) [file pone.0243797.s002.docx]

S1 Table. The Newcastle-Ottawa Scale of individual study

| Study | Selection | | | | Comparability | Outcome | | | NOS |
| --- | --- | --- | --- | --- | --- | --- | --- | --- | --- |
|  | Representativeness of the exposed cohort | Selection of the non exposed cohort | Ascertainment  of exposure | Demonstration that outcomes was not present at start of study | Comparability on the basis of the design or analysis | Assessment of outcome | Adequate follow-up duration | Adequate follow-up rate | Overall score |
| Armstrong 1997 [21] | 0 | 1 | 1 | 1 | 2 | 1 | 1 | 1 | 8 |
| Lin 2010 [22] | 0 | 1 | 1 | 1 | 2 | 1 | 1 | 1 | 8 |
| Akinci 2011 [23] | 1 | 1 | 1 | 1 | 2 | 1 | 1 | 1 | 9 |
| Aziz 2011 [24] | 1 | 1 | 1 | 1 | 2 | 1 | 1 | 1 | 9 |
| Tunccan 2012 [25] | 1 | 1 | 1 | 1 | 2 | 1 | 1 | 1 | 9 |
| Ulcay 2014 [26] | 1 | 1 | 1 | 1 | 2 | 1 | 1 | 1 | 9 |
| Saltoglu 2015 [27] | 0 | 1 | 1 | 1 | 2 | 1 | 1 | 1 | 8 |
| Pickwell 2015 [28] | 1 | 1 | 1 | 1 | 2 | 1 | 1 | 1 | 9 |
| Tabur 2015 [29] | 0 | 1 | 1 | 1 | 2 | 1 | 1 | 0 | 7 |
| Quilici 2016 [30] | 0 | 1 | 1 | 1 | 2 | 1 | 1 | 0 | 7 |
| Uysal 2017 [31] | 0 | 1 | 1 | 1 | 2 | 1 | 1 | 0 | 7 |
| Cervantes-García 2017 [32] | 0 | 1 | 1 | 1 | 2 | 1 | 1 | 1 | 8 |
| Ferreira 2018 [33] | 0 | 1 | 1 | 1 | 2 | 1 | 1 | 0 | 7 |
| Musa 2018 [34] | 0 | 1 | 1 | 1 | 2 | 1 | 1 | 0 | 7 |
| Khalfallah 2018 [35] | 0 | 1 | 1 | 1 | 2 | 1 | 1 | 1 | 8 |
| Peled 2019 [36] | 0 | 1 | 1 | 1 | 2 | 1 | 1 | 1 | 8 |
| Guo 2019 [37] | 0 | 1 | 1 | 1 | 2 | 1 | 1 | 0 | 7 |
| Jeyaraman 2019 [38] | 0 | 1 | 1 | 1 | 2 | 1 | 1 | 1 | 8 |
| Ugwu 2019 [39] | 0 | 1 | 1 | 1 | 2 | 1 | 1 | 0 | 7 |
| Sayiner 2019 [40] | 0 | 1 | 1 | 1 | 2 | 1 | 1 | 0 | 7 |
| Aziz 2020 [41] | 1 | 1 | 1 | 1 | 2 | 1 | 1 | 1 | 9 |
| Gandhi 2020 [42] | 1 | 1 | 1 | 1 | 2 | 1 | 1 | 1 | 9 |
